# TRSRD: a database for research on risky substances in tea using natural language processing and knowledge graph-based techniques

**DOI:** 10.1093/database/baad031

**Published:** 2023-05-09

**Authors:** Yongmei Wang, Peng Wang, Yongheng Zhang, Siyi Yao, Zhipeng Xu, Youhua Zhang

**Affiliations:** Anhui Provincial Engineering Laboratory for Beidou Precision Agriculture Information, School of Information and Computer, Anhui Agricultural University, 130 Changjiangxilu, Heifei, Anhui 230036, P.R.China; Anhui Provincial Engineering Laboratory for Beidou Precision Agriculture Information, School of Information and Computer, Anhui Agricultural University, 130 Changjiangxilu, Heifei, Anhui 230036, P.R.China; Anhui Provincial Engineering Laboratory for Beidou Precision Agriculture Information, School of Information and Computer, Anhui Agricultural University, 130 Changjiangxilu, Heifei, Anhui 230036, P.R.China; Anhui Provincial Engineering Laboratory for Beidou Precision Agriculture Information, School of Information and Computer, Anhui Agricultural University, 130 Changjiangxilu, Heifei, Anhui 230036, P.R.China; Anhui Provincial Engineering Laboratory for Beidou Precision Agriculture Information, School of Information and Computer, Anhui Agricultural University, 130 Changjiangxilu, Heifei, Anhui 230036, P.R.China; Anhui Provincial Engineering Laboratory for Beidou Precision Agriculture Information, School of Information and Computer, Anhui Agricultural University, 130 Changjiangxilu, Heifei, Anhui 230036, P.R.China

## Abstract

During the production and processing of tea, harmful substances are often introduced. However, they have never been systematically integrated, and it is impossible to understand the harmful substances that may be introduced during tea production and their related relationships when searching for papers. To address these issues, a database on tea risk substances and their research relationships was constructed. These data were correlated by knowledge mapping techniques, and a Neo4j graph database centered on tea risk substance research was constructed, containing 4189 nodes and 9400 correlations (e.g. research category-PMID, risk substance category-PMID, and risk substance-PMID). This is the first knowledge-based graph database that is specifically designed for integrating and analyzing risk substances in tea and related research, containing nine main types of tea risk substances (including a comprehensive discussion of inclusion pollutants, heavy metals, pesticides, environmental pollutants, mycotoxins, microorganisms, radioactive isotopes, plant growth regulators, and others) and six types of tea research papers (including reviews, safety evaluations/risk assessments, prevention and control measures, detection methods, residual/pollution situations, and data analysis/data measurement). It is an essential reference for exploring the causes of the formation of risk substances in tea and the safety standards of tea in the future.

**Database URL** http://trsrd.wpengxs.cn

## Introduction

Tea is an aromatic beverage made from the processed leaves of the tea tree and is one of the most popular beverages in the world (1). Tea has unique flavors (2) and is rich in beneficial chemicals such as catechins, theaflavins, and 5-nethylglutamine (3). The polyphenols in tea can promote human health (4), and the tea has medicinal value (5). Some studies have shown that drinking tea every day has been shown to reduce weight (6, 7), prevent cancer (8), and regulate intestinal flora (9) and may also reduce the risk of cardiovascular disease (10). The food safety standards of the importing country are a crucial factor that can impact the tea trade flow (11, 12). As research into the food safety considerations of tea is conducted, more harmful substances that may be introduced during tea production are being identified. In addition to pesticide exposure in tea production (13, 14) and heavy metal risks (15), there are also residues of other chemicals (16, 17), such as 9,10-anthraquinone (18) and perchlorate (19). Nevertheless, a vast amount of information regarding hazardous chemicals in tea is dispersed throughout various literature sources, which poses difficulties for researchers in searching for relevant papers and integrating knowledge into a systematic framework. Consequently, this situation impedes the progress of research. Therefore, it is essential to establish a complete database for research on tea risk substances.

In order to solve the above problems, we have constructed a database that catalogs tea risk substances and their corresponding research relationships. We used text mining technology (20) to collect papers that discuss harmful substances in tea. We used text abstracts and text classification to filter and classify papers. We classified all papers into nine categories of hazardous substances and six categories of research, to distinguish papers discussing various harmful substances and identify the specific research focus on such substances. This enables researchers to easily search and comprehend relevant information (21). Subsequently, the categorized data underwent syntactic analysis and chemical entity extraction to independently identify the chemical substances discussed in each paper. These data were then linked through knowledge mapping techniques (22) correlation to create a Neo4j database containing 4189 nodes and 9400 correlations, which allows the researcher to better discover hidden relationships between studies (23).

Our main contributions to this study are as follows.

- Papers on harmful substances in tea were consolidated, summarized, and classified. The chemical substances discussed in these papers were independently identified through syntactic analysis and entity extraction. The papers have been comprehensively collected and compiled for the first time.
- A workflow has been developed from document mining to text classification to entity extraction, linking the scattered knowledge in different documents using knowledge graphs.
- This database provides a visual view of data and research data, which can be used to find relationships between studies on harmful substances in tea for reference and research citation.

## Steps and methods

**Figure 1.**
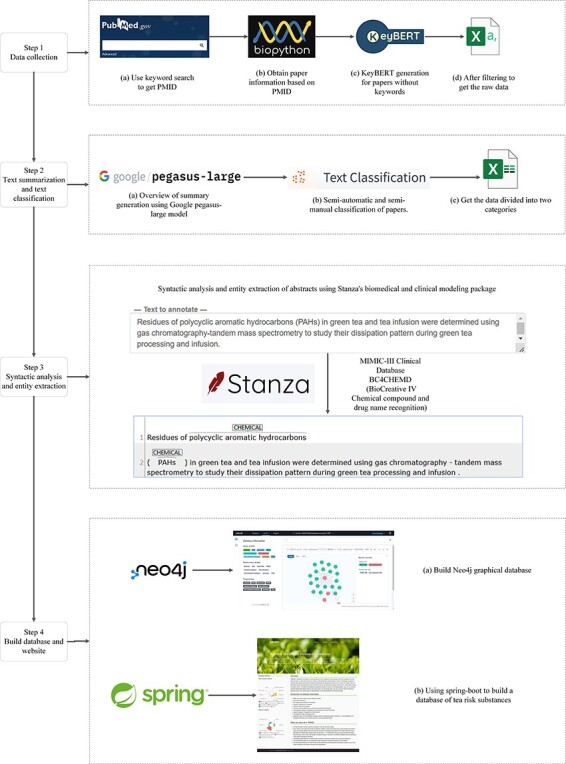
The Neo4j database construction process.

This study classified the papers by extracting essential information from the literature abstracts. Each paper was labeled with its risk substance category (divided into nine categories) and its research category (divided into six categories). The text of the literature abstracts was syntactically analyzed according to the classification results, and chemical entities were separated from them, resulting in 955 chemical substances. The processed data were then used as PubMed Unique Identifier (PMID) to establish the corresponding relationships. This resulted in the construction of a Neo4j graph database of tea risk substances and their research relationships. The construction process is shown in Figure 1.

## Data collection

First, all relevant literature PMIDs were obtained from PubMed using keywords such as tea, pesticide, and food safety. Subsequently, PMIDs were obtained from the PubMed database using Biopython (24) to extract the desired literature’s titles, DOIs, keywords, and abstracts. At this stage, papers without keywords or abstracts were removed from the dataset. For the remaining papers without keywords but with an abstract, KeyBERT (25) was used to generate six keywords, resulting in 5177 usable results. Finally, the initial data were obtained by retrieving and filtering information from the titles, keywords, and abstracts and deleting papers irrelevant to tea. The valuable information was selected here to facilitate later overview and classification operations on this information without the effect of useless information (26).

### Text summarization and classification

After obtaining the initial filtered data, the long abstracts were compressed by Google’s pegasus-large model (27) for paragraph comprehension, information compression (28), and language generation (29) to produce an abstract overview of only a few sentences, the purpose of this step is to compress the long abstracts into short, fluent, readable texts that retain the most salient information (30). The title, keywords, abstract, and summary are utilized collectively to classify each paper into two categories: the risk substance category (comprising of inclusion pollutants (comprehensive discussion), heavy metals, pesticides, environmental pollutants, mycotoxins, microorganisms, radioactive isotopes, plant growth regulators, and others) and the paper’s research category (including review, safety evaluation/risk assessment, prevention and control measures, detection methods, residual/pollution situation, and data analysis/data measurement). If the keywords and abstracts contain the content of a risky substance category and are of a single type, they will be assigned directly to that category, otherwise, they will be classified as pollutants (comprehensive discussion). As there is currently no classification model for tea research, the remaining papers were manually classified, and those that were not manually classified were checked twice at a later stage to ensure correctness. Table 1 shows the risk substance categories and their classification criteria. Table 2 presents the classification criteria for the paper study categories.

**Table 1.** Risk substance categories and their classification criteria

Risk substance category	Classification criteria
Pollutants (comprehensive discussion)	This item is used when two or more different pollutants are included.
Heavy metals	This item is used when only heavy metals are contained.
Pesticides	Only contains pesticides or insecticides for this item.
Environmental pollutants	This item is used when only environmental pollutants are contained.
Mycotoxins	This item is used when only mycotoxins are contained.
Microorganisms	This item is used when only microorganisms are contained.
Radioactive isotopes	This item is used when only radioactive isotopes are contained.
Plant growth regulators	This item is used when only plant growth regulators are contained.
Others	This is the item if it is not one of the above categories.

**Table 2.** Research categories of dissertations and their classification criteria

Research category	Classification criteria
Review	Summary overview of hazardous substances
Safety evaluation/risk assessment	Assess the risk of the hazardous substance or study its harm to humans
Prevention and control measures	Reduce the residue of the hazardous substance or avoid the use of the hazardous substance
Detection methods	Innovative detection methods for this hazardous substance
Residual/pollution situation	Study the residual pattern of the pollutant or investigate the pollution of a certain area by the pollutant
Data analysis/data measurement	Large-scale data collection and analysis of pollutants at a site

### Syntactic analysis and entity extraction

After classifying the paper, in order to find out the chemical substances, we need to first analyze the syntactic structure of the sentences (31) in the abstract, and then, meaningful information is identified from the split syntactic structure by named entity recognition (32). With the research and development of natural language processing, natural language processing models trained on specific datasets are also often used in the biomedical field. Biomedical natural language processing is often used for word sense disambiguation, named entity recognition, information extraction, and relation extraction (33).

To ensure the accuracy of named entity recognition, we chose Stanza (34), a neural natural language processing package customized for biomedical text processing, using the biomedical and clinical syntactic analysis and named entity recognition models provided in Stanza. For the abstracts after classification, chosen a syntactic analysis pipeline trained on the MIMIC clinical dataset (35) and a named entity recognition model pre-trained on the BC4CHEMD corpus (36). After separating the chemical entities, these substances are grouped into corresponding papers to build a database.

### Build database and website

For database selection, we chose the Neo4j graph database in order to store the connections and relationships between data in more flexible numbers with data elements. In Neo4j, the data are stored like a whiteboard, which makes Neo4j flexible compared to other graph databases. While doing the above advantages, the Neo4j graph database also has better performance (37).

Finally, these data are aggregated, building nodes and relationships using the Neo4j graph database to create a Tea Risk Substance Research Database (TRSRD). TRSRD allows researchers to easily access and analyze the data we have collected and processed.

## Results

### Knowledge mapping for tea risk substance research

**Figure 2.**
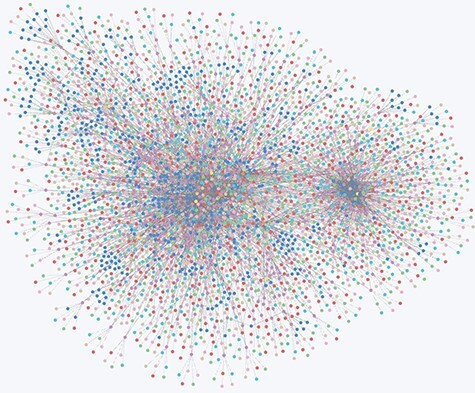
Overview of all node relationships in the Neo4j database.

With 4189 nodes and 9400 associations, TRSRD divides all the literature into nine risk substance categories and six paper research categories and also extracts 955 different tea risk substances from the classified papers to build a knowledge graph, so that researchers can quickly visualize the different hazardous substances in tea without having to do a lot of searching and summarizing in the field. This allows researchers to quickly visualize the different studies on harmful substances in tea without having to do extensive searches in the field, which helps in the referencing and citation of research. A complete visualization of the data in the database is shown in Figure 2.

### Neo4j database management website

**Figure 3.**
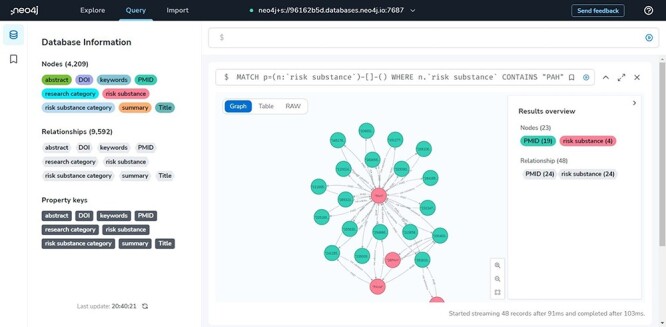
Neo4j database management site showcase.

The developers mainly use this site to maintain and check the data, and when the data need to be updated, new data can be redeployed from this site. It is also possible to view the visualization of all the data in the database as shown in Figure 3.

### Website (TRSRD) page

**Figure 4.**
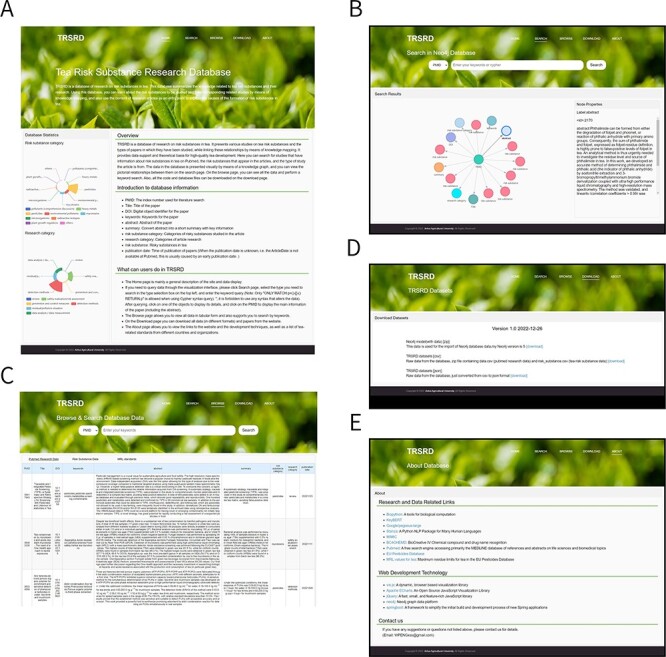
All pages of the TRSRD are displayed. (A) The homepage of the website provides a brief introduction of the website content and presents data statistics. (B) The knowledge graph search page of the website allows for visual exploration of the data. (C) The data browsing page of the website enables viewing and searching of the data in tabular format. (D) The download page of the website allows for direct downloading of the database data. (E) The about page of the website lists the data sources and other related database links.

The TRSRD website provides a friendly and intuitive interactive interface that allows users to browse the site’s introduction, search the Neo4j database (returned as visible results), view data (in tabular form), and download all data. We built the site using a front- and back-end separation, using Spring Boot to connect to the Neo4j database as the back end of the TRSRD site and native HyperText Markup Language with a JavaScript framework to build the front end of the TRSRD site. Figure 4 shows the details of the TRSRD.

### Website main page

The website’s main page shows an introduction and overview of TRSRD, while the database statistics on the left-hand side show the distribution of risk substance data and research papers in the database and are presented in a Nightingale rose diagram (Figure 4A).

### Search and browse page

The search page on the website enables users to input specific keywords and obtain visible search results, facilitating intuitive data retrieval and query. Clicking on a node allows users to view the < id> of the node and the corresponding value (Figure 4B). The browse page displays all the data in a table format and searches for the specified keywords (Figure 4C). On the browse page, you can view all temporarily included paper data (sorted by publication date), extracted risk substance data and the Maximum Residue Levels standards specified by different countries or organizations.

### Download and About page

The download page lets users download the data format they need in Neo4j database import format, CSV table format, and JavaScript Object Notation format (Figure 4D). On the About page, users can view links to research and data related to the site and links to technical web development, which can be accessed by clicking on the corresponding website. In addition, in the contact section below, users can get our email (Figure 4E).

## Conclusion

As tea becomes increasingly popular in various countries, more and more people are becoming aware of it. At the same time, research into the safety of tea has gradually increased. However, papers on tea quality and safety have never been systematically integrated. As a result, researchers cannot quickly and easily search for harmful substances in their desired field. In this paper, we have constructed a database centered on tea risk substance research by filtering and classifying existing tea research data through natural language processing and performing named entity extraction. Researchers can use TRSRD to better understand the risk substances in tea and the corresponding research, which is an essential reference for exploring the formation of risk substances in tea and future safety standards for tea.

As tea research continues to develop, we will continue to understand the harmful substances in tea and improve its safety standards. Our current work may still have shortcomings, such as the classification of chemical substances is not precise enough, not for the whole industry chain to make a more detailed way of classification. In addition, the model used on named entity identification does not achieve perfect identification and still requires manual screening of data. In the future, we will continue to monitor the latest research results in this field, incorporate more hazardous substances into the TRSRD, enrich the classification in the TRSRD, improve the classification criteria, and make the TRSRD a reference and citation platform among tea researchers.

## Data Availability

All required data are contained in the database website and are available for download by all users. The database website address is http://trsrd.wpengxs.cn.
